# 

**DOI:** 10.1192/bjb.2025.10178

**Published:** 2026-08

**Authors:** Rebecca Lawrence

**Affiliations:** Psychiatry, NHS Lothian, Edinburgh, UK.

**Figure:**
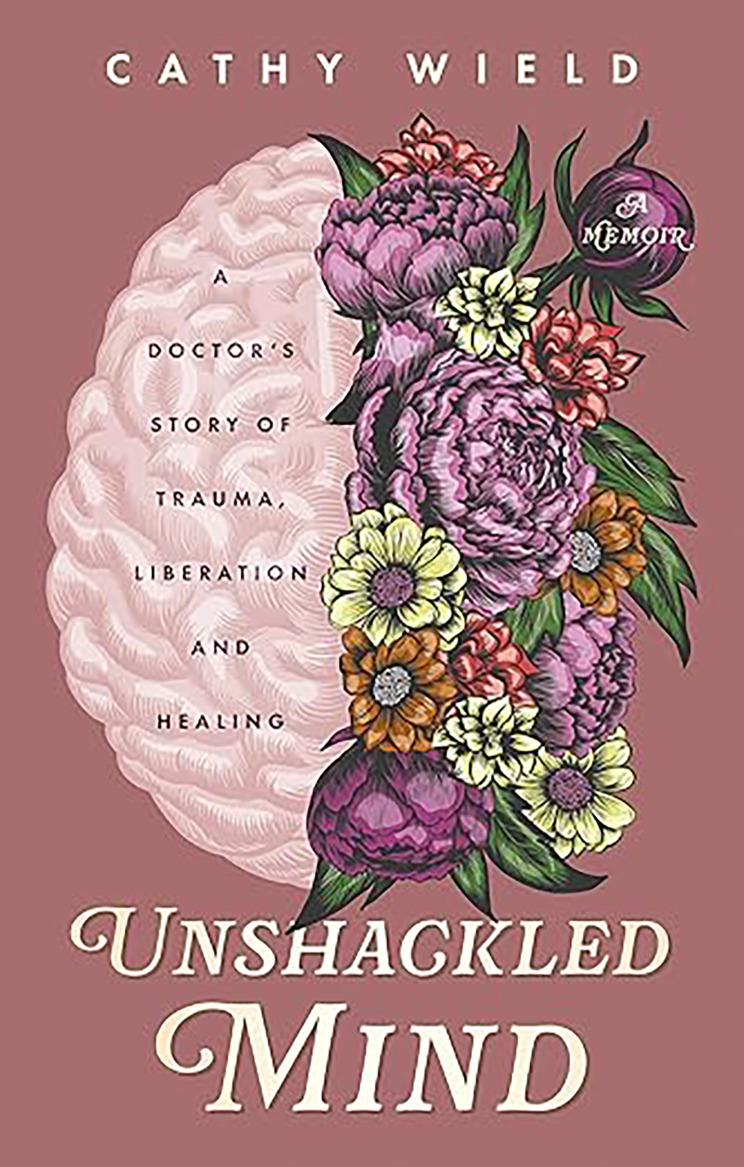

*Unshackled Mind*, by Cathy Wield, has left its author ‘free at last from the shackles of psychiatry, free from the shackles of evangelicalism and free from the shackles placed on a young child sent away to boarding school’. It is an unusual and compelling book, in that the first half retells a story previously told by the same author, of her experience of severe depression, hospitalisation, treatment with multiple drugs, electroconvulsive therapy (ECT) and, ultimately, psychosurgery. *Life after Darkness: A Doctor’s Journey through Severe Depression* (CRC Press, 2005) was a book that made me question psychiatric treatments and hospitalisation, I think rightly so, and this new book raises other questions.

There are threads that spiral through both books, and views about these will differ. Early trauma often coexists with mental illness and its treatments, and these will influence each other. However, this doesn’t necessarily imply causation. It is easy to hear the echoes of parental separation in Wield’s anguished attempts to communicate with her psychiatrists; but it is difficult, after so many years, to fully appreciate the levels of concern felt by professionals, as well as the risks they were trying to manage.

There is an interesting parallel between the lightbulb moment cure following psychosurgery and her sudden realisation, talking with her therapist years later, that depression might not have been her diagnosis. Wield’s determination is ferocious and commendable, although she herself recognises that this may have developed as a coping mechanism following early events. Her work in accident and emergency departments, and with the homeless, demonstrates an appreciation of the wide diversity of contributing factors experienced by those with mental distress, but acceptance of all the multifaceted influences on her own life at times seems lacking. She rejects psychiatry totally – in Denver, she is ‘mortified by the idea of a psychiatric assessment’, even in different circumstances.

I was concerned by her confident statement that there is not good evidence for the use of psychotropic drugs and ECT, but I would agree that it may be best not to persist with them if ineffective. I also agree that tapering off these drugs can be very hard, and can lack appropriate support. Another comment, made several times, is that psychotropic drugs do not cure mental illness. This may well be true, but relief of symptoms is important. This is mirrored in other medical specialties, and does not prove that mental illness does not exist.

Ultimately, what I found hardest when reading this was her description of herself as ‘Emily’, writing in the third person. I can appreciate that it might have been hard to write in the first person, but, as someone who had read the first book, it felt at times as though she was describing someone else. Perhaps, in a way, she was.

